# Delayed-onset descemet membrane detachment after uneventful cataract surgery treated by corneal venting incision with air tamponade: a case report

**DOI:** 10.1186/s12886-016-0212-6

**Published:** 2016-04-04

**Authors:** Harsimran Kaur Bhatia, Rakesh Gupta

**Affiliations:** Shreya Eye Centre, D-163, Surajmal Vihar, Delhi, India

**Keywords:** Descemet membrane detachment, Delayed-onset DMD, Pneumatic descemetopexy, Air tamponade, Expansile gases, Corneal venting incision, Supra-descemet fluid, Case report

## Abstract

**Background:**

Descemet membrane detachment (DMD) is a significant complication noted during or early after cataract surgery. Review of literature revealed a few cases of delayed-onset DMD with presentation ranging from weeks to months after cataract surgery but most of them were treated with pneumatic descemetopexy and a few ended in penetrating keratoplasty. We report this case, to highlight the usefulness of corneal venting incision with air tamponade in late-onset DMD cases not responding to pneumatic descemetopexy.

**Case presentation:**

A retrospective case review of a 66 year old male who presented with diminution of vision in right eye 17 days after uneventful cataract surgery was done. Visual acuity in this eye was 20/200 at presentation. DMD was noted 3 days later (approximately 3 weeks post-operatively) and Anterior Segment Optical Coherence Tomography & Scheimpflug imaging were done in view of diffuse corneal edema. Pneumatic descemetopexy was attempted thrice (twice with SF6, once with air) over a week’s span with limited success at re-attaching the DM. Finally, corneal venting incision with air tamponade was done resulting in egress of supra-descemet’s fluid and DM appeared apposed to stroma. Bandage contact lens (BCL) was applied at the end of the procedure. DM was seen attached the next day. Corneal edema cleared completely in 1 week. Best corrected visual acuity (BCVA) at 6 weeks follow-up was 20/30.

**Conclusion:**

Delayed-onset DMD should be considered as a differential diagnosis in cases with late-onset corneal edema post-cataract surgery. Anterior segment Optical Coherence Tomography (AS-OCT) and Scheimpflug Imaging are useful tools in cases with dense corneal edema. Corneal venting incision with air tamponade is an option in cases where methods like pneumatic descemetopexy fail.

## Background

Descemet membrane detachment is a serious complication of surgical procedures involving anterior chamber manipulation [1]. It is most commonly encountered during cataract surgery and diagnosis is made intra-operatively in 50 % of the cases [2]. Rarely, it can develop late in the post – op period varying from weeks to months [3–6]. Clinical presentation is that of decreased vision associated with corneal edema. If left untreated, edema may persist, leading to corneal decompensation and vision loss. The conventional treatment strategies include injection of air, viscoelastic or expansile gas and penetrating keratoplasty in decompensated corneas [1–4, 6]. The presence of supra-descemet’s fluid may however, hinder the apposition of DM to stroma and fail repeated attempts of pneumatic descemetopexy [7]. We report one such unusual case of delayed onset DMD, presenting with decreased vision 17 days after uneventful phacoemulsification. Following three unsuccessful attempts of pneumatic descemetopexy, corneal venting incision with drainage of supra-descemet’s fluid and air tamponade resulted in successful DM reattachment and good visual outcome.

## Case presentation

**Fig. 1:**
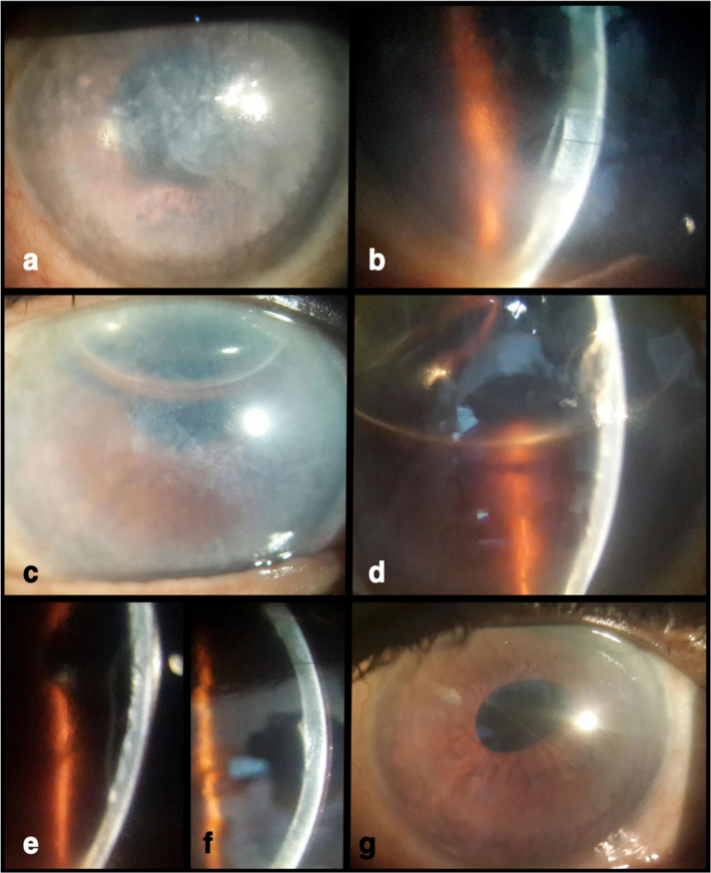
**a** Diffuse corneal edema, prominent centrally (POD-17). **b** Central DMD (POD-20). **c** SF6 bubble in situ; Superiorly stromal edema present; clear cornea inferiorly (POD-21). **d** SF6 bubble (second injection), corneal edema with detached DM (POD-22). **e** Central DMD; decreased corneal stromal edema (POD-27). **f** Clear cornea (1 week after corneal venting incision with tamponade). **g** DM attached completely (1 week after corneal venting incision with tamponade)

**Fig. 2:**
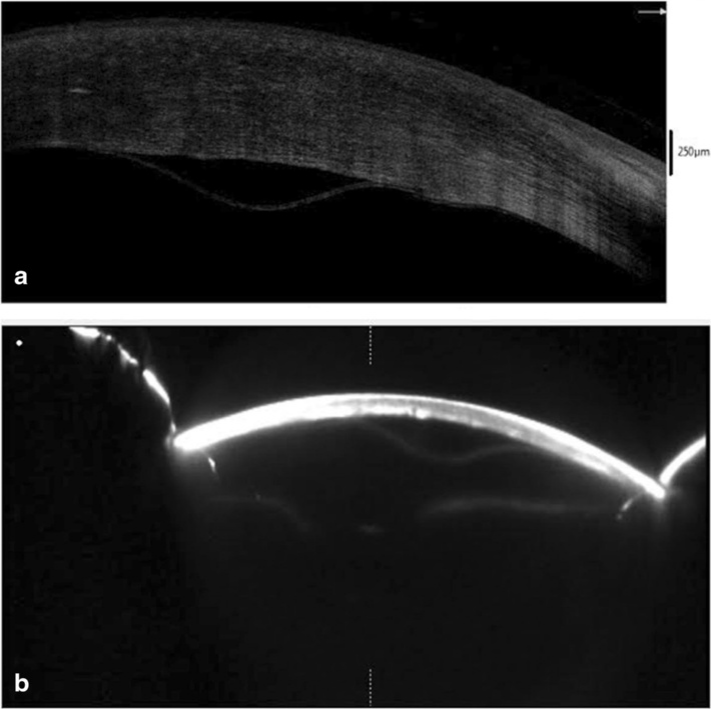
**a** AS-OCT image: Central DMD with increased corneal thickness. **b** Scheimpflug imaging: Central DMD

**Fig. 3:**
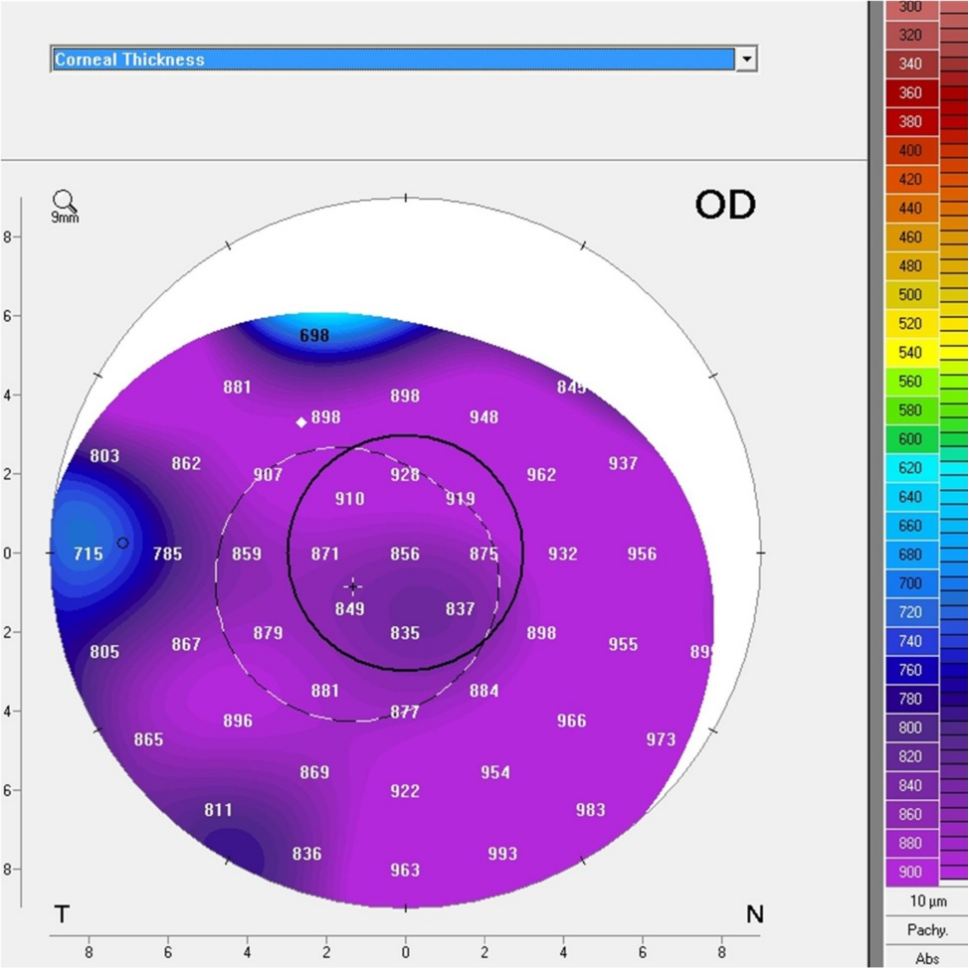
Scheimpflug imaging: Pachymetry at the apex - 856 μm; Thinnest pachymetry - 698 μm

A 66 year old male presented with reduced vision in right eye 17 days after uneventful phacoemulsification with intraocular lens implantation at our centre. Uncorrected visual acuity (UCVA) in right eye was 20/200 and left eye was 20/80. Slit lamp examination revealed right eye diffuse corneal stromal edema more pronounced centrally with descemet’s folds Fig. 1a. The anterior chamber was deep and quiet and intraocular lens was in situ. On fundus examination, details were hazily discerned due to stromal edema. Left eye had immature senile cataract and no other positive findings were noted. The intraocular pressure was 14 mm Hg in both eyes. Conservative management in the form of Prednisolone acetate 1 % eyedrop, Hypertonic Saline 6 % eye ointment (Hypersol-6 Eye Ointment) and Homatropine eyedrop were started. Review of previous records revealed that right eye cornea was clear and BCVA was 20/30 at 1 week postoperative visit. Patient followed up three days later (Post-op day-20) and a DMD was noted Fig. 1b. Intracameral SF_6_ 20 % (0.2 ml) was injected under topical anesthesia. Oral Acetazolamide was also started. Next day (POD-21), DM was well apposed, inferiorly cornea was clear but superiorly corneal edema persisted Fig. 1c. The very next day (POD-22), DM was seen re-detaching and Injection SF6 was repeated Fig. 1d. The DM was still detached the next day (POD-23), hence pneumatic descemetopexy was repeated but this time with air. Four days later (POD-27), corneal edema was seen resolving, improving the uncorrected visual acuity to 20/120 but central DM cleft was still present Fig. 1e. This time corneal venting incision with air tamponade was performed. Under topical anesthesia, a venting incision was made at the site of maximum stromal edema, using MVR blade. Air was injected to fill almost 2/3 of the anterior chamber to provide tamponade. Corneal massaging was done in a centripetal fashion (from periphery to centre) with rounded edges of a blunt forceps and egress of supra-descemet’s fluid was seen. This was continued till DM appeared apposed to corneal stroma. BCL was applied at the end of the procedure. DM was re-attached the next day and corneal edema cleared completely in a week’s time Fig. 1f and g. Patient was followed up at 2 weeks and at 6 weeks postoperatively the BCVA was 20/30. Both AS-OCT and Scheimpflug imaging revealed the central location of DMD Fig. 2a and b. Pachymetry at the apex was 856 μm and thinnest pachymetry was 698 μm as seen on Scheimpflug imaging Fig. 3.

## Discussion

DMD was first described by Weve in 1927. Of all the procedures involving anterior chamber entry, it is reported most commonly after cataract surgery. The predisposing factors include hazy cornea, shallow anterior chamber and hypotony. Intra-operatively, use of blunt instruments for entry, oblique entry and anterior shelved corneal wounds, inadvertent injection of air, saline, antibiotics or viscoelastic between the stroma and descemet’s membrane, pinching of DM with aspiration cannula, hooking of IOL haptic into DM increase the chances of this complication [8]. Marcon et al have attributed increased referrals of DMD to the increasing use of clear corneal incisions [1]. Rarely, DMD can occur in the intermediate to late post-op period, after uncomplicated surgery [3–6]. In the case discussed, DMD was noted 20 days (approximately 3 weeks) after uneventful cataract surgery. Although the entry wound was clear corneal, which in itself is a predisposing factor, the central location of detachment and clear cornea at previous visits ruled out the role of faulty wound location or construction and any kind of intra-op trauma to DM. Morkin et al have reported one such case of late DMD 11 months after cataract surgery and discussed the possibility of trauma as the triggering factor [6]. In the case discussed, there was neither a history of eye rubbing nor any other trauma. The existence of underlying predisposing anatomical factors can be considered especially in cases with bilateral involvement [4]. Pre-existing poor endothelial counts as a significant risk factor has been put forth by Ti et al. [9] In our case, although specular microscopy was not done, the absence of corneal guttae pre-operatively gave an impression of healthy endothelium. Similarly, Kansal et al have suggested abnormal fibrillary stromal adhesion to Descemet’s membrane as the possible cause [10]. Genetic predisposition in the form of dysfunctional anchoring protein BIGH3 (due to mutation of TGFBI gene) has been postulated by Hirano et al. [11] Although literature suggests various theories, the exact pathophysiology of delayed onset DMD still remains poorly understood due to lack of concrete evidence.

Management is largely dependent on intracameral injections of expansile gases like C_3_F_8_ or SF_6_ given the ease and good success rate of the procedure [1, 2, 4, 6]. We attempted pneumatic descemetopexy twice with SF_6_ without a positive result. Anticipating a similar outcome, air was injected at third place and as expected detached DM failed to settle. Finally, corneal venting incision with air tamponade was done resulting in DM attachment. These corneal venting incisions have variable applications in DMD cases. Menezo et al reported one of the earliest cases suggesting the role of corneal paracentesis with air tamponade for DMD after cataract surgery [12]. Ghaffariyeh et al successfully attempted supra-descemet fluid drainage with corneal venting incisions for DMD after phacoemulsification without the use of any air/gas tamponade [7]. Looking at the success of this technique we tried it in our case and achieved complete descemet’s re-attachment with good visual outcome. Literature also describes the role of these incisions for drainage of interface fluid following Descemet’s stripping endothelial keratoplasty (DSEK) [13]. The underlying principle of this technique is to drain the fluid entrapped in the supra-descemet’s space that otherwise prevents the apposition of DM to stroma even after pneumatic tamponade.

## Conclusion

This discussion is meant to highlight the delayed-onset DMD which should be considered as a differential diagnosis in cases with late-onset corneal edema post-cataract surgery. An AS-OCT or a Scheimpflug imaging is warranted in post-operative cases with dense corneal edema to pick up any DMD and also differentiate it from cases of pseudophakic or aphakic bullous keratopathy (PBK/ABK) . Apart from pneumatic descemetopexy, corneal venting incision with air tamponade is an option that can be tried in non-resolving cases. More studies with larger sample sizes need to be done to explain the mechanism of delayed-onset DMD and diagnose the cases at risk before contemplating surgery. Early diagnosis and intervention promises excellent visual outcome and avoids vision threatening complications in such cases.

### Ethics committee

The approval was not required as the data has been analyzed in a retrospective manner and has no effect on treatment of the patient.

### Consent for publication

Written informed consent was obtained from the patient for publication of this Case report and any accompanying images.

## Abbreviations

AS-OCT: Anterior segment-optical coherence tomography
ABK: Aphakic bullous keratopathy
BCVA: Best corrected visual acuity
DM: Descemet membrane
DMD: Descemet membrane detachment
DSEK: Descemet stripping endothelial keratoplasty
PBK: Pseudophakic bullous keratopathy
SF_6_: Sulphur hexafluoride
TGFBI: Transforming growth factor, beta-induced
UCVA: Uncorrected visual acuity
